# Autopsies and Asymptomatic Patients During the COVID-19 Pandemic: Balancing Risk and Reward

**DOI:** 10.3389/fpubh.2020.595405

**Published:** 2020-11-09

**Authors:** Matteo Nioi, Pietro Emanuele Napoli, Maurizio Fossarello, Ernesto d'Aloja

**Affiliations:** ^1^Forensic Medicine Unit, Department of Clinical Sciences and Public Health, University of Cagliari, Cagliari, Italy; ^2^Department of Surgical Science, Eye Clinic, University of Cagliari, Cagliari, Italy

**Keywords:** COVID-19 pandemic, COVID-19 autopsy, COVID-19 autopsy asymptomatic, autopsy safety, COVID-19 tests, SARS-CoV-2 cadaveric persistence, COVID-19 HCWs

Although we have gained much knowledge of coronavirus disease 2019 (COVID-19), its pathogenesis needs further investigation. Autopsies seem fundamental to confirming the Severe Acute Respiratory Syndrome-Coronavirus 2 (SARS-CoV-2) transmission pathways, pathogenetic mechanisms, and natural history.

The air and contact transmission routes of SARS-CoV-2 have been well-established, but further confirmatory work is needed on the oral–fecal, hematic, and ocular routes (1). Also, some aspects of the natural history of SARS-CoV-2 remain unclear. For example, what are the interindividual characteristics that determine the severity of COVID-19 among those infected with the virus?

The mechanisms by which SARS-CoV-2 infection leads to extrapulmonary manifestations are also unclear. Based on the symptoms affecting the gustatory and olfactory system, some authors have hypothesized an underlying neurogenic mechanism.

The pathogenesis of the important symptoms affecting the vascular system should also be defined. Some studies have suggested that it is these effects on the vascular system that are responsible for the thrombo-embolic events leading to the terminal phase of COVID-19 (2).

However, one of the most important unsolved questions concerns the very high number of asymptomatic patients. This category includes individuals who are positive for SARS-CoV-2 but with no clinical or radiological manifestations of COVID-19. Their percentage is estimated, in some preliminary studies, to be approximately 17.9% in a “closed” system. However, the real number of asymptomatic subjects is likely largely underestimated given the pandemic nature of COVID-19 and the low number of tests performed. Health policies have provided for a low number of autopsies and swabbing in subjects for whom there was no suspicion of COVID-19. This prevents an understanding of whether positivity in SARS-CoV-2 can lead to subclinical pathological changes. Clarifying whether there are pathological or genetic factors (3) that can explain why some individuals are not susceptible to the virus or, when testing positive, do not develop the pathology is of fundamental importance.

Greater clarity on these issues would surely help when developing treatment and prophylaxis strategies for COVID-19.

These strategies could undoubtedly have therapeutic importance and could help guide weighted choices about how best to protect public health; asymptomatic patients are a potential source of contagion (4).

The existence of asymptomatic SARS-CoV-2 infection is an intriguing puzzle; it does represent a potential danger for those who perform autopsies.

Although transmission by asymptomatic patients is a crucial point in the ongoing pandemic, the guidelines for performing autopsies continue to underestimate the biological risk posed by such patients. The importance of the topic is relevant. In Italy, for example, several law firms are proposing lawsuits against health workers who treated the victims of COVID-19: this phenomenon will inevitably translate into an increase in autopsies on infected corpses (5).

The current indications concern deceased individuals “who have died of suspected or confirmed COVID-19” or in which the presence of the disease is considered “possible” (6).

Although several authors have proposed alternative solutions that might lower the risk of infection, these solutions do not guarantee that all cases will be intercepted. In the current context, the discoveries on the SARS-CoV-2 virus are extremely fast, and there is a risk that “provisional” indications conceived in the first phase may be outdated or require the introduction of an implementation in a few weeks or months. The indications given for the first phase were those used before the pandemic for diseases with a high risk of infection.

Thus, the recommendations included the use of autopsy rooms with a biological safety level of at least 3 (BSL-3). Another recommendation is an adequate use of personal protective equipment (PPE) including a surgical suit, headgear, visor, FFP3 mask, and waterproof gloves that extend up to the forearm with targeted use of oropharyngeal swabs. Another suggestion concerned particular sectoral techniques to be used in suspected cases such as manual opening of the skull and block sampling of heart and lungs. Among the most complete recommendations produced so far are those of Aquila et al. who hypothesize the possibility of carrying out a swab even on individuals in whom there is no suspicion of COVID-19 with consequent adjustments to the procedure in the event of a positivity. However, even this guide does not consider the pre-autoptic phase (judicial inspection), the post-autoptic phase (transport from the sectoral room to the burial place), and the analytical limits linked to the swab (7, 8).

The sensitivity of reverse transcriptase-PCR (RT-PCR) tests of throat and pharyngeal swabs has been estimated at around 70%. (9) Even the most sensitive serological tests are unable to intercept positivity in the early stages of infection (10).

Finally, it should be noted that these tests have not been validated on postmortem samples, and the persistence time of the virus in affected corpses remains—to our knowledge—undetermined.

To conclude, we believe that the widespread and systematic execution of autopsies will contribute to our knowledge of COVID-19. However, caution is needed, and for now, all corpses should be treated as a source of SARS-CoV-2, regardless of swab or serological test results.

## Author Contributions

MN, Ed'A, and PN conceived of the presented idea. MF developed the theory. MN wrote the manuscript in consultation with Ed'A, PN, and MF. All authors contributed to the article and approved the submitted version.

## Conflict of Interest

The authors declare that the research was conducted in the absence of any commercial or financial relationships that could be construed as a potential conflict of interest.
